# Characterization of the USDA *Cucurbita pepo*, *C. moschata*, and *C. maxima germplasm* collections

**DOI:** 10.3389/fpls.2023.1130814

**Published:** 2023-03-13

**Authors:** Christopher O. Hernandez, Joanne Labate, Kathleen Reitsma, Jack Fabrizio, Kan Bao, Zhangjun Fei, Rebecca Grumet, Michael Mazourek

**Affiliations:** ^1^ Department of Agriculture Nutrition and Food Systems, University of New Hampshire, Durham, NH, United States; ^2^ Plant Genetic Resource Conservation Unit, United States Department of Agricultural Research Service, Geneva, NY, United States; ^3^ North Central Regional Plant Introduction Station, Iowa State University, Ames, IA, United States; ^4^ Plant Breeding and Genetics, Cornell University, Ithaca, NY, United States; ^5^ Boyce Thompson Institute, Cornell University, Ithaca, NY, United States; ^6^ U.S. Department of Agriculture-Agriculture Research Service, Robert W. Holley Center for Agriculture and Health, Ithaca, NY, United States; ^7^ Department of Horticulture, Michigan State University, East Lansing, MI, United States

**Keywords:** germplasm, genotyping-by-sequencing, GWAS, diversity, *Cucurbita*

## Abstract

The *Cucurbita* genus is home to a number of economically and culturally important species. We present the analysis of genotype data generated through genotyping-by-sequencing of the USDA germplasm collections of *Cucurbita pepo*, *C. moschata*, and *C. maxima*. These collections include a mixture of wild, landrace, and cultivated specimens from all over the world. Roughly 1,500 - 32,000 high-quality single nucleotide polymorphisms (SNPs) were called in each of the collections, which ranged in size from 314 to 829 accessions. Genomic analyses were conducted to characterize the diversity in each of the species. Analysis revealed extensive structure corresponding to a combination of geographical origin and morphotype/market class. Genome-wide associate studies (GWAS) were conducted using both historical and contemporary data. Signals were observed for several traits, but the strongest was for the bush (*Bu*) gene in *C. pepo*. Analysis of genomic heritability, together with population structure and GWAS results, was used to demonstrate a close alignment of seed size in *C. pepo*, maturity in *C. moschata*, and plant habit in *C. maxima* with genetic subgroups. These data represent a large, valuable collection of sequenced *Cucurbita* that can be used to direct the maintenance of genetic diversity, for developing breeding resources, and to help prioritize whole-genome re-sequencing.

## 1 Introduction

The *Cucurbitaceae* (Cucurbit) family is home to a number of vining species mostly cultivated for their fruits. This diverse and economically important family includes cucumber (*Cucumis sativus*), melon (*Cucumis melo*), watermelon (*Citrullus lanatus*), and squash (*Cucurbita ssp.*) (Ferriol and Picó, 2008). Like other cucurbits, squash exhibit diversity in growth habit, fruit morphology, metabolite content, disease resistance, and have a nuanced domestication story (Paris and Brown, 2005; Chomicki et al., 2020). The genomes of *Cucurbita ssp.* are small (roughly 400 Mb), but result from complex interactions between ancient genomes brought together through an allopolyploidization event (Sun et al., 2017). These factors make squash an excellent model for understanding the biology of genomes, fruit development, and domestication. Within *Cucurbita*, three species are broadly cultivated: *C. maxima*, *C. moschata*, and *C. pepo* (Ferriol and Picó, 2008). Few genomic resources have been available for these species; although, draft genomes and annotations, along with web-based tools and other genomics data are emerging (Yu et al., 2023). Already, these resources have been used to elucidate the genetics of fruit quality, growth habit, disease resistance, as well as to increase the efficiency of cucurbit improvement (Montero-Pau et al., 2017; Zhong et al., 2017; Kaźmińska et al., 2018; Xanthopoulou et al., 2019; Wu et al., 2019a; Hernandez et al., 2020). However, there has yet to be a comprehensive survey of the genetic diversity in the large diverse *Cucurbita* germplasm panels maintained by the USDA within the National Plant Germplasm System.

Germplasm collections play a vital role in maintaining and preserving genetic variation. These collections can be mined by breeders for valuable alleles. They can also be used by geneticists and biologists for mapping studies (McCouch et al., 2020). Like many other orphan and specialty crops, there has been little effort put into developing community genetic resources for squash and other cucurbits. The Cucurbit Coordinated Agricultural Project (CucCAP project) was established to help close the knowledge gap in cucurbits (Grumet et al., 2021). This collaborative project aims to provide genomics resources and tools that can aid in both applied breeding and basic research. The genetic and phenotypic diversity present in the USDA watermelon, melon, and cucumber collections has already been explored as part of the CucCAP project, partially through the sequencing of USDA germplasm collections and development of core collections for whole-genome sequencing (Wang et al., 2018; Wu et al., 2019b; Wang et al., 2021). The diverse specimens of the USDA squash collections have yet to be well characterized at the genetic level. An understanding of squash diversity requires an appreciation of the elaborate system used to classify squash.

The classification system used in squash is complex. Squash from each species can be classified as either winter or summer squash depending on whether the fruit is consumed at an immature or mature stage, the latter is a winter squash (Loy, 2004). Squash are considered ornamental if they are used for decoration, and some irregularly shaped, inedible ornamental squash are called gourds. Gourds, however, include members of *Cucurbita* as well as some species from *Lagenaria*, and as a result, not all gourds are squash (Paris, 2015). Many squash are known as pumpkins; the pumpkin designation is a culture dependent colloquialism that can refer to Jack O’ Lantern types, squash used for desserts or, in some Latin American countries, to eating squash from *C. moschata* known locally as Calabaza (Ferriol and Picó, 2008). Cultivars deemed as pumpkins can be found in all widely cultivated squash species. Unlike the previous groupings, morphotypes/market classes are defined within species. For example, a Zucchini is reliably a member of *C. pepo* and Buttercups are from *C. maxima*. Adding to the complexity of their classification, the *Cucurbita* species are believed to have arisen from independent domestication events and the relationships between cultivated and wild species remain poorly understood (Kates et al., 2017).

*C. pepo* is the most economically important of the *Cucurbita* species and is split into two different subspecies: *C. pepo* subsp. *pepo* and *C. pepo* subsp. *ovifera* (Xanthopoulou et al., 2019). Evidence points to Mexico as the center of origin for *pepo* and southwest/central United States as the origin of *ovifera*. The progenitor of *ovifera* is considered by some to be subsp. *ovifera* var. *texana*, whereas subsp. *fraterna* is a candidate progenitor for *pepo* (Kates et al., 2017). Europe played a crucial role as a secondary center of diversification for subsp. *pepo*, but not subsp. *ovifera* (Lust and Paris, 2016). Important morphoptypes of *pepo* include Zucchini, Spaghetti, Cocozelle, Vegetable Marrow, and some ornamental pumpkins. *C. pepo* subsp. *ovifera* includes summer squash from the Crookneck, Scallop, and Straightneck group, and winter squash such as Delicata and Acorn (Paris et al., 2012).

The origin of *C. moschata* is more uncertain than *C. pepo*; it is unclear whether *C. moschata* has its origin in South or North America (Chomicki et al., 2020). Where and when domestication occurred for this species is also unknown; however, it is known that *C. moschata* had an India-Myanmar secondary center of origin where the species was further diversified (Sun et al., 2017). *C. moschata* plays an important role in squash breeding as it is cross-fertile to various degrees with *C. pepo* and *C. maxima*, and can thus be used as a bridge to move genes across species (Sun et al., 2017). Popular market classes of *C. moschata* include cheese types like Dickinson, which is widely used for canned pumpkin products, Butternut (Neck) types, Japonica, and tropical pumpkins known as Calabaza (Ferriol and Picó, 2008).

*C. maxima* contains many popular winter squash including Buttercup/Kabocha types, Kuri, Hubbard, and Banana squash (Ferriol and Picó, 2008). This species also sports the world’s largest fruit, the giant pumpkin, whose fruit are grown for competition and can reach well over 1000 Kg (Savage et al., 2015). Although this species exhibits a wide range of phenotypic diversity in terms of fruit characteristics, it appears to be the least genetically diverse of the three species described (Kates et al., 2017). *C. maxima* is believed to have a South American origin, and was likely domesticated near Peru, with a secondary center of domestication in Japan and China (Nee, 1990; Sun et al., 2017).

In this study, we set out to characterize the genetic diversity present in the USDA *Cucurbita* germplasm collections for *C. pepo*, *C. moschata*, and *C. maxima*. We present genotyping-by-sequencing (GBS) data from each of these collections, population genomics analysis, results from genome-wide association studies (GWAS) using historical and contemporary phenotypes, and suggest a core panel for re-sequencing.

## 2 Materials and methods

### 2.1 Plant materials and genotyping

All available germplasm were requested from USDA cooperators for *C. maxima* (534 accessions from Geneva, NY), *C. moschata* (314 accessions from Griffin, GA), and *C. pepo* (829 accessions from Ames, IA). Seeds were planted in 50-cell trays and two 19 mm punches of tissue (approximately 80-150 mg) was sampled from the first true leaf of each seedling. DNA was extracted using Omega Mag-Bind Plant DNA DS kits (M1130, Omega Bio-Tek, Norcross, GA) and quantified using Quant-iT PicoGreen dsDNA Kit (Invitrogen, Carlsbad, CA). Purified DNA was shipped to Cornell’s Genomic Diversity Facility for GBS library preparation using protocols optimized for each species. Libraries were sequenced at either 96, 192, or 384-plex on the HiSeq 2500 (Illumina Inc., USA) with single-end mode and a read length of 101 bp.

### 2.2 Variant calling and filtering

SNP calling was conducted using the TASSEL-GBS V5 pipeline (Glaubitz et al., 2014). Tags produced by this pipeline were aligned using the default settings of the BWA aligner (Li and Durbin, 2009). Raw variants were filtered using BCFtools (Danecek et al., 2021). Settings for filtering SNPs were as follows, minor allele frequency (MAF) ≥ 0.05, missingness ≤ 0.4, and biallelic. Nine genotypes were removed based on missing data and preliminary PCA results in *C. maxima*. One genotype was removed from extitC. pepo (See **Supplemental Info S1**). Variants were further filtered for specific uses as described below.

### 2.3 Population genomics analysis

ADMIXTURE (Alexander and Lange, 2011), which uses a model-based approach to infer ancestral populations (*k*) and admixture proportions in a given sample, was used to explore population structure in each dataset. ADMIXTURE does not model linkage disequilibrium (LD); thus, marker sets were further filtered to obtain SNPs in approximate linkage equilibrium using the “–indep-pairwise” option in PLINK (Purcell et al., 2007) with *r* ^2^ set to 0.1, a window size of 50 SNPs, and a 10 SNP step size. All samples labeled as cultivars or breeding material were removed from the data prior to running ADMIXTURE. These samples were removed to prevent structure created through breeding from appearing as ancestral populations. Ancestral populations were then assigned to cultivars after training on data without the cultivars using the program’s projection feature. Cross-validation was used to determine the best *k* value for each species. Briefly, ADMIXTURE was run with different values (1-20) and the cross-validation error was reported for each *k*. The most parsimonious *k* value with minimal cross-validation error was chosen for each species.

Principal components analysis (PCA) was used as a model-free way of determining population structure. PCA was conducted using SNPRelate (Zheng et al., 2012) on the same LD-pruned data used by ADMIXTURE.

Linkage disequilibrium was calculated in each germplasm panel using VCFtools (Danecek et al., 2011) with the settings “–geno-r2 –ld-window 1000”. Filtered, but not pruned, data were used for the LD calculation.

### 2.4 Analysis of phenotypic data

Historical data were obtained from the USDA Germplasm Resources Information Network (GRIN; www.ars-grin.gov) for *C. maxima*, *C. pepo*, and *C. moschata*. All duplicated entries were removed for qualitative traits, where categories are mutually exclusive, leaving only samples with unique entries for analysis. Phenotypic data from two traits, adult and nymph squash bug damage, in *C. pepo* were transformed using the boxcox procedure. Contemporary phenotypic data were collected from a subset of the *C. pepo* collection grown in the summer of 2018 in Ithaca, NY. Field-grown plants were phenotyped for vining bush habit at three different stages during the growing seasons to confirm bush, semi-bush or vining growth habit. Plants that had a bush habit early in the season but started to vine at the end of the season were considered semi-bush.

### 2.5 GWAS

Variant data were filtered to MAF ≥ 0.05 and missingness ≤ 0.2, and then imputed prior to association analysis. LinkImpute (Money et al., 2015), as implemented by the TASSEL (Bradbury et al., 2007) “LDKNNiImputatioHetV2Plugin” plugin was used for imputation with default settings. Any data still missing after this process were mean imputed. The GENESIS (Gogarten et al., 2019) R package, which can model both binary and continuous traits, was used for conducting the associations. All models included the first two PCs of the marker matrix as fixed effects and modeled genotype effect (*u*) as a random effect distributed according to the kinship (*K*) matrix ( $u∼N(0,\sigma_{u}^{2}\text{K})$ ). Binary traits were modeled using the logistic regression feature of GENESIS. The kinship matrix was calculated using A.mat from rrBLUP (Endelman, 2011) with mean imputation.

### 2.6 Genomic heritability

An estimate of genomic heritability ( $h_{G}^{2}$ ) (de los Campos et al., 2015) was calculated for all ordinal and quantitative traits using an equivalent model to what was used for GWAS, but without fixed effects. Variance components from the random genetic effect ( $\sigma_{u}^{2}$ ) and error ( $\sigma_{e}^{2}$ ) were then used to calculate the heritability as $h_{G}^{2}=\frac{\sigma_{u}^{2}}{\sigma_{u}^{2}+\sigma_{e}^{2}}$ .

### 2.7 Syntenty of Bu putative region in C. pepo and C. maxima

A candidate gene for dwarfism (bush phenotype), *Bu*, in *C. maxima* was elucidated by a previous study and was named Cma_004516 (Zhang et al., 2015). Gene ID in the Cucurbit Genomics Database corresponding to Cma_004516 was identified by using the BLAST tool to align primer sequences used for RT-QPCR in the previous study (Zhang et al., 2015) against the *C. maxima* reference genome. The synteny analysis was done by using the Synteny Viewer tool and evaluating *C. maxima*’s chromosome 3 with *C. pepo*’s chromosome 10 and searching for an ortholog to the candidate gene. The physical position of the *C. pepo* ortholog was identified by searching the gene using the Search tool. All tools used in the analysis can be found on the Cucurbit Genomics Database at cucurbitgenomics.org/v2/.

### 2.8 Identification of a core collection

Subsets representative of each panel’s genetic diversity were identified using GenoCore (Jeong et al., 2017) with the filtered SNP sets. The GenoCore settings were “-cv 99 -d 0.001”.

## 3 Results

### 3.1 Genotyping

**Figure 1:**
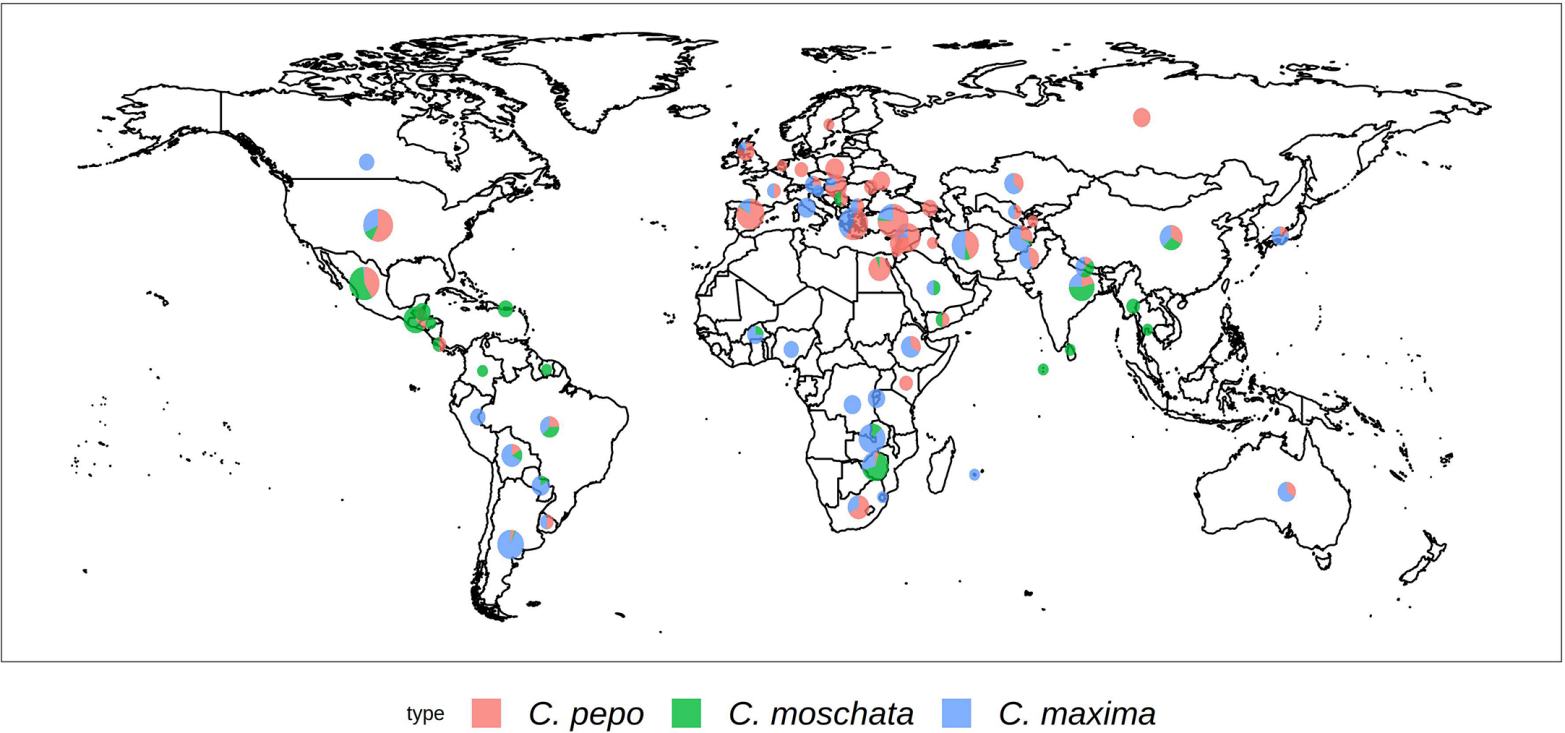
Geographical distribution of the USDA *Cucurbita* ssp. collection. The size of the pie chart is scaled according to the number of accessions. Sector areas correspond to the proportion of the three.

Each *Cucurbita* ssp. collection was genotyped using the GBS approach. The collections comprised 534 accessions for *C. maxima*, 314 for *C. moschata*, and 829 for *C. pepo*. **Figure 1** shows the geographical distribution of accessions broken down by species. *C. maxima* and *C. moschata* constitute the majority of accessions collected from Central and South America, whereas *C. pepo* accessions are more prevalent in North America and Europe. *C. pepo* had the highest number of raw SNPs (88,437) followed by *C. moschata* (72,025) and *C. maxima* (56,598). After filtering, *C. pepo* and *C. moschata* had a similar number of SNPs, around 30,000, whereas *C. maxima* had an order of magnitude fewer filtered SNPs (1599). This discrepancy may be an artifact of using PstI, a rarer base-cutter previously optimized for GBS of *C. maxima* [46], rather than ApeKI which was used for *C. pepo* and *C. moschata*. The number and distribution of SNPs across each chromosomes is shown in **Table 1**. Maps of SNP distribution for each species are shown in **Supplemental Figure S1**.

**Table 1:** Distribution and number of raw and filtered SNPs per chromosome for each species.

	*C. pepo*		*C. moschata*		*C. maxima*	
Chromosome	Raw	Filtered	Raw	Filtered	Raw	Filtered
0	12498	2550	2708	546	1501	132
1	7497	2831	3890	1468	4185	121
2	5153	2049	3661	1538	2101	55
3	4875	1943	3472	1499	2201	51
4	4598	1982	6880	2553	5703	106
5	4045	1628	2716	887	3115	46
6	3871	1384	3262	1159	3035	92
7	3129	1222	2668	969	2705	62
8	3875	1583	2348	810	2391	61
9	3766	1390	3106	995	2750	84
10	3585	1488	3550	1327	2297	52
11	3227	1216	4336	1830	3713	131
12	3089	1163	3711	1330	2026	47
13	3434	1350	3106	1280	2131	82
14	3543	1291	4753	1929	4317	100
15	2640	960	3564	1321	2662	58
16	3088	1060	2933	1107	2058	100
17	2994	1175	2885	1096	2195	86
18	3053	1258	3341	1316	1826	46
19	3381	1340	2638	990	1793	46
20	3096	1155	2497	903	1893	41
**TOTAL**	**88437**	**32018**	**72025**	**26853**	**56598**	**1599**

### 3.2 Population structure and genetic diversity

**Figure 2:**
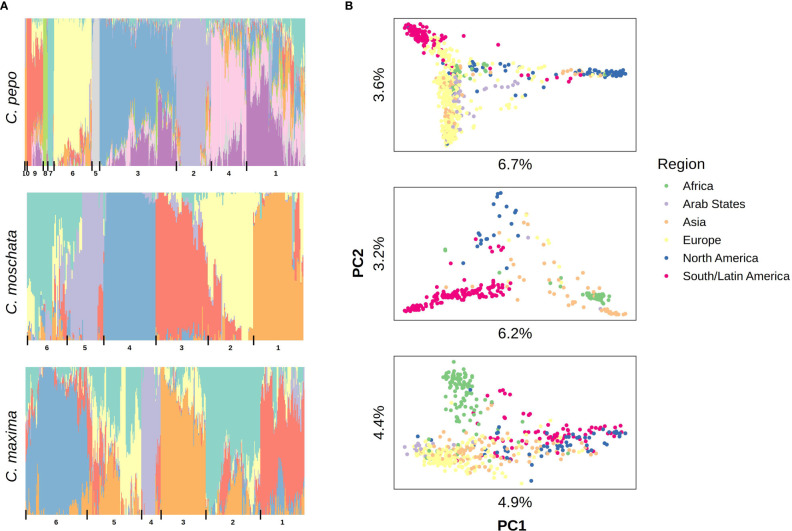
Population structure results aligned vertically by species. **(A)** Admixture plots: each stacked barplot represents an accession colored by proportion of inferred ancestral population. Groups based on hierarchical clustering are delimited by vertical bars and labeled with numbers along the bottom. **(B)** Plots of the first two principal components (PC) of accessions colored by region, variation explained by PCs is labeled on each axis.

**Figure 3:**
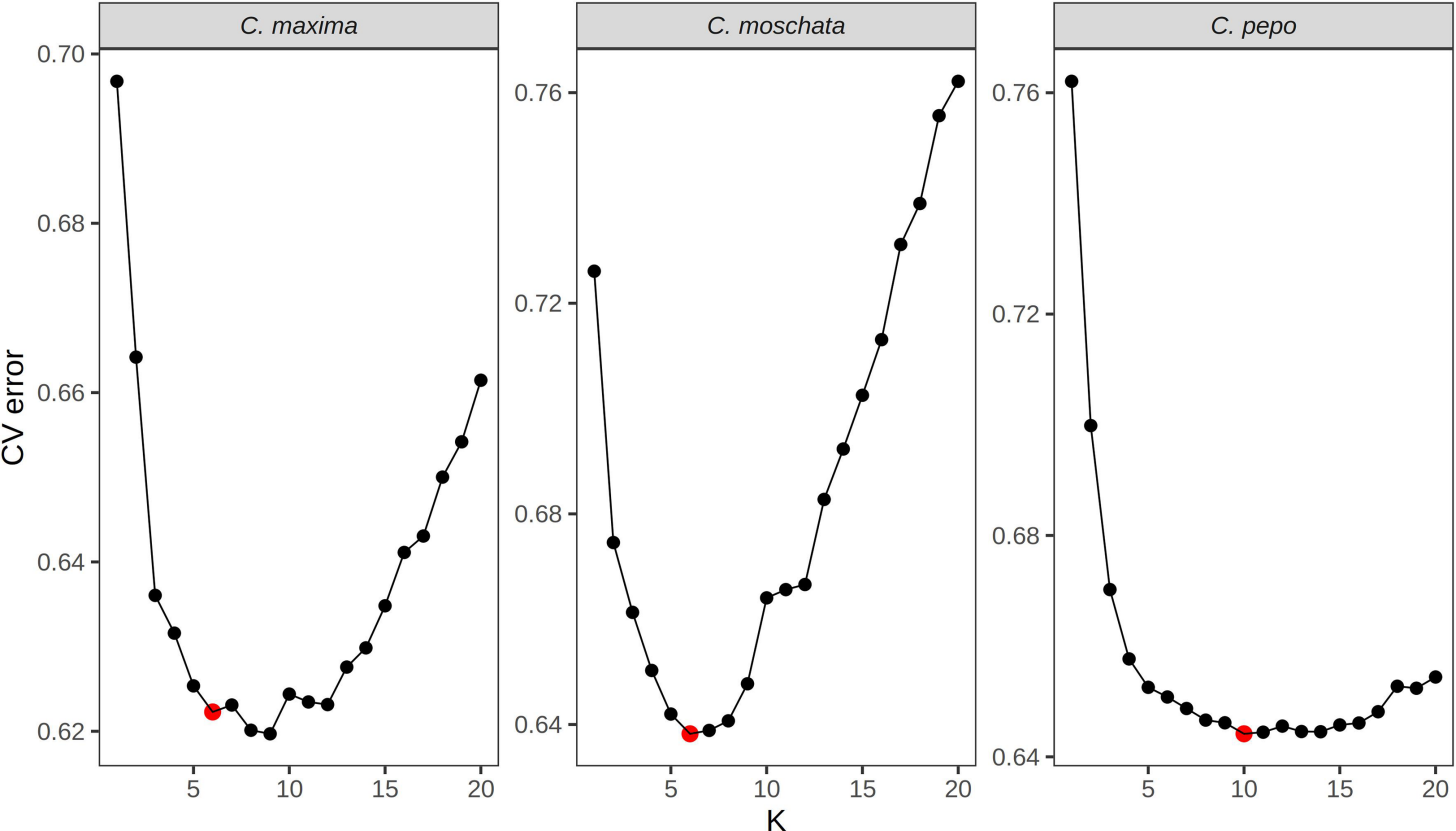
Cross-validation error plots used to pick the optimum *k* value for admixture analysis. The *k* value that balances minimizing cross-validation error and parsimony was chosen for the final analysis. The chosen *k* is labeled with a red point.

Filtered SNPs were used for population structure analysis. Available geographical, phenotypic, and other metadata were retrieved from GRIN and were used to help interpret structure results. Results from model-based admixture analysis are shown in **Figure 2A**. These data support 10 ancestral groups (K=10) in *C. pepo*, 6 in *C. moschata*, and 6 in *C. maxima*. The number of groups was based on the cross-validation error output of ADMIXTURE shown in **Figure 3**. For *C. pepo* and *C. moschata*, a clear minimum was reached. The optimal *k* for both roughly agreed with the number of known morpho-market classes and/or subspecies. In *C. maxima*, a local minimum was reach at *k* = 6 followed by a slight decrease after *k* = 8. For the sake of parsimony, and consistency with known morpho-market classes in *C. maxima*, a *k* of 6 was chosen. Population structure was driven mostly by geography, except in *C. pepo* where the presence of different subspecies was responsible for some of the structure. Commonalities among structure groups are described in **Table 2**. The first two principal components (PCs) of the marker data are shown in **Figure 2B**. As with the model-based analysis, PCA showed geography as a main driver of population structure with accessions being derived from Africa, the Arab States, Asia, Europe, North America, and South/Latin America. PC1 in *C. pepo* separates *C. pepo* subsp. *ovifera*, which have a North American origin, from subsp. *pepo*.

**Table 2:** Commonalities among accessions in each group, most groupings are dictated by geography.

*C. pepo*	*C. moschata*	*C. maxima*
1 Mixed Group; Many from Spain, Turkey, and Syria	Mostly from Mexico	Mixed; Primarily from South America and Asia
2 Wild subsp. *ovifera* var. *texana* and var. *ozarkana*; North American	Mostly Mexico and Guatemala	Mostly Mexico and Guatemala
3 Majority from Turkey	Mostly from Mexico	Mostly from North Macedonia
4 Majority from North Macedonia	Mostly from Africa	Mostly from Argentina
5 Majority from Egypt	Mostly from India	Mostly Turkey, Iran, Afghanistan
6 Majority from Mexico	Mixed origin Europe and Americas; Many similar to cheese or neck type	Mostly from Africa
7 Majority from Syria		
8 Majority from Pakistan and Afghanistan		
9 Majority from Spain		
10 Wild subsp. *fraterna*; Central American		

**Figure 4:**
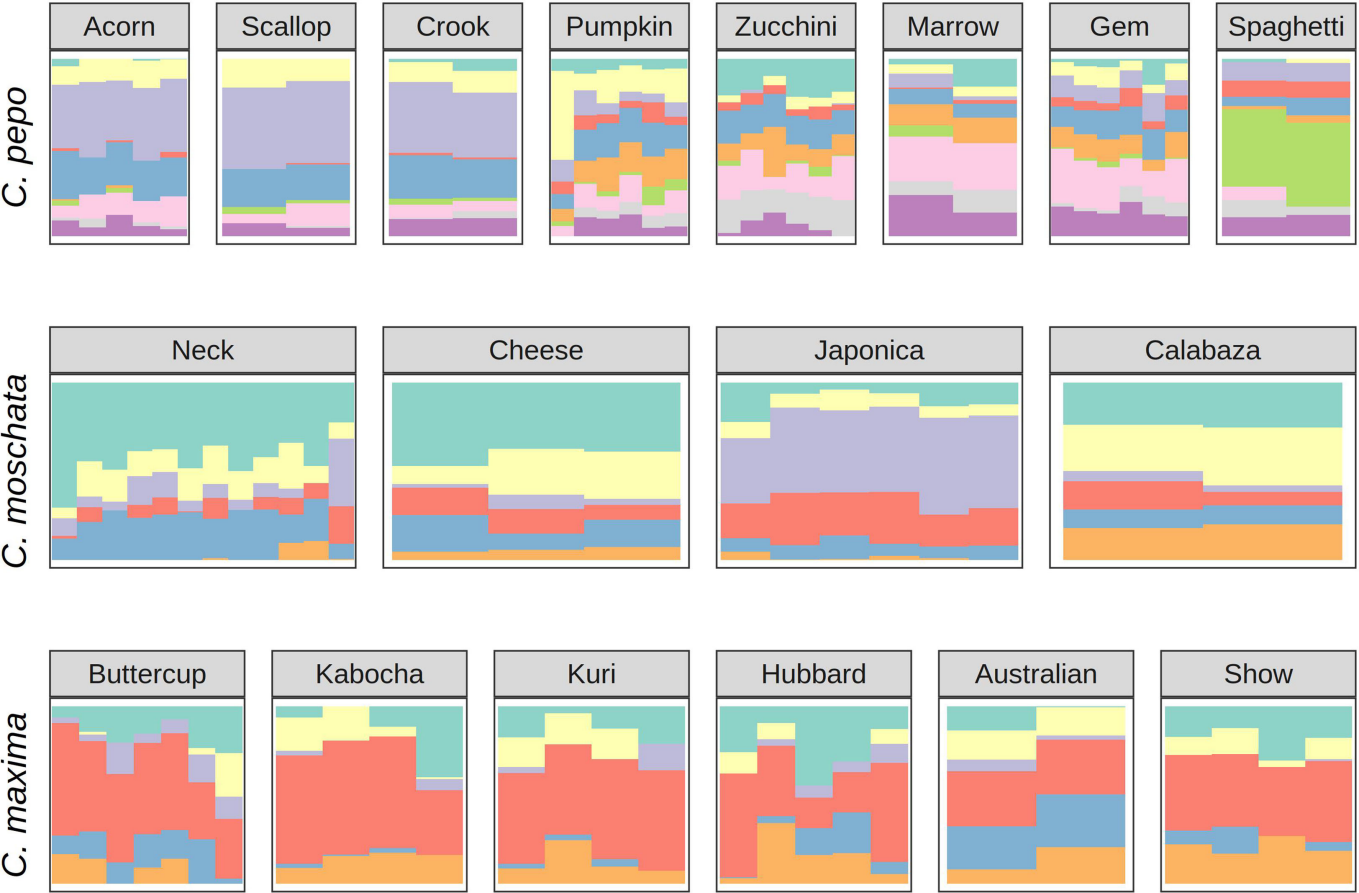
Ancestry coefficients projected on cultivars from each species. Results are shown grouped by market/varietal class. Colors correspond to the groups in **Figure 3A**.

Ancestry proportions from admixture analysis were projected onto cultivars/market types identified in the accessions. Cultivars were grouped according to known market class within species to help identify patterns in ancestry among and between market classes. Key market types identified in accessions from *C. pepo* include Acorn, Scallop, Crook, Pumpkin (Jack O’ Lantern), Zucchini, Marrow, Gem, and Spaghetti; Neck, Cheese, Japonica, and Calabaza in *C. moschata*; and Buttercup, Kobocha, Hubbard, and Show (Giant squash) in *C. maxima*. These groupings are shown in **Figure 4**. In general, members of each market class exhibit similar ancestry proportions. In *C. pepo*, market classes from the two different subspecies had distinct ancestry patterns. For example, Acorn, Scallop and Crook market classes are all from subsp. *ovifera* and all of these classes had similar ancestry proportions with roughly 20% of ancestry from the wild *ovifera*. In contrast, market classes within subsp. *pepo* had a small percentage of ancestry from wild *ovifera* and more ancestry in common with European and Asian accessions. With *C. moschata*, Neck, Cheese, and Calabaza market classes showed every similar ancestry patterns, whereas the Japonica class was more distinct. Relative to the *C. pepo* and *C. moschata*, the *C. maxima* cultivars were less differentiated from one another.

**Figure 5:**
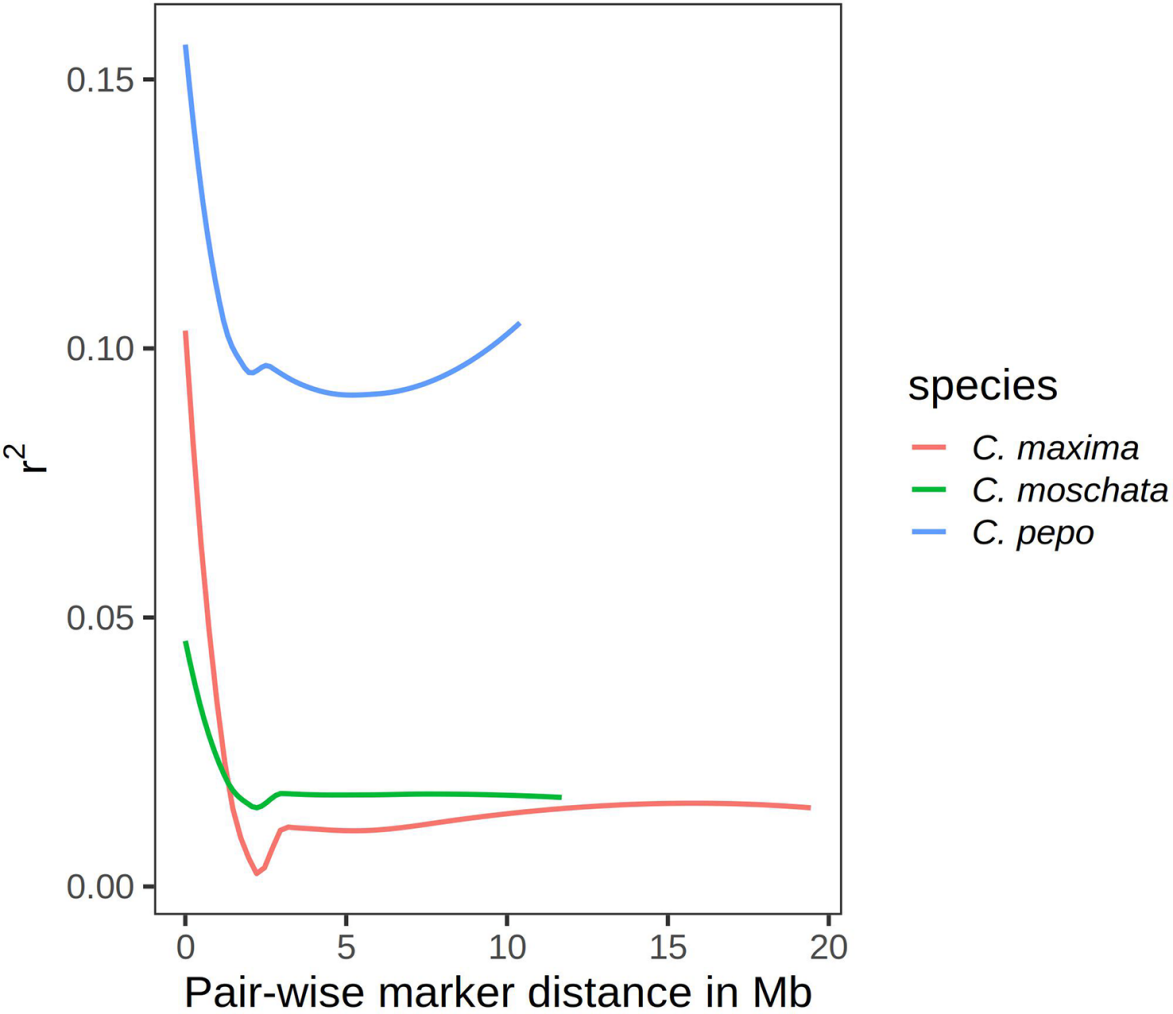
Curves showing *r* ^2^ value as a measure of LD on the y-axis and pair-wise distance between markers in megabases on the x-axis.

Results from linkage-disequilibrium analysis are shown in **Figure 5**. Similar trends are seen across species. In general, LD decays to zero once the distance between markers reaches more than 2 megabases (Mb). *C. pepo* maintains a higher LD, with an average R-squared between markers of 0.1 even beyond 2 Mb.

### 3.3 Analysis of phenotypic data

All historical phenotypic data from GRIN were compiled for analysis. Only traits with ≥ 100 entries were considered for further analysis. Filtering resulted in 26 traits for *C. pepo*, 5 for *C. moschata* and 16 for *C. maxima*. Traits spanned fruit and agronomic-related characteristics, as well as pest resistances. The number of records for a given trait ranged from 108 to 822, with an average of 270. Fruit traits included fruit width, length, surface color and texture, and flesh color and thickness. Agronomic data included plant vigor and vining habit, and several phenotypes related to maturity. Pest-related traits included susceptibility to cucumber beetle and squash bug in *C. pepo* and Watermelon mosaic virus (WMV) and powdery mildew (PM) in *C. maxima*. **Supplemental Figure S2** shows the distribution for each quantitative trait.

**Figure 6:**
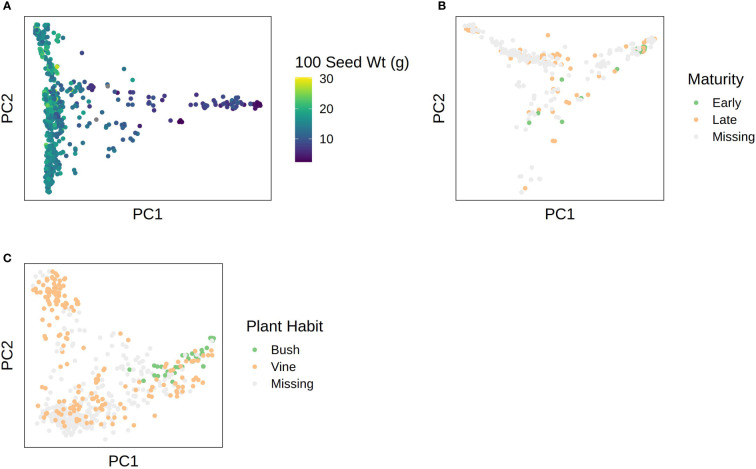
PCA plots with phenotypes superimposed over them for: **(A)** 100 seed weight in *C. pepo*; **(B)** maturity in *C. moschata*; **(C)** plant habit in *C. maxima*.

Phenotypic data were superimposed over the first two PCs in each species to visualize correspondence between population structure and phenotype. Results are shown in **Figure 6**. In *C. pepo*, seed size was almost completely confounded with subspecies, with subsp. *ovifera* having mostly small seeds and subsp. *pepo* having larger seeds (**Figure 6A**). In *C. moschata*, maturity was confounded with population structure (**Figure 6B**). In *C. maxima*, plant habit was confounded with population structure (**Figure 6C**).

### 3.4 Genomic heritability

An estimate of genomic heritability was calculated for all quantitative and ordinal traits and is shown in **Table 3**. In *C. pepo*, seed weight and morphological traits such as fruit length and width had very high (> 0.7) heritability estimates. Disease and insect resistance traits had lower heritabilites from 0.181-0.228. Trends were similar in both *C. moschata* and *C. maxima*, with *C. maxima* having lower heritability estimates across the board.

**Table 3:** Descriptive data for each trait including trait type, number of data points for each trait, a brief trait description, and an estimate of genomic heritability $h_{G}^{2}$ .

Species	Trait	N	Type	Description	H^2^G
***C. pepo***					
	seed wt	827	Quantitative	Weight of 100 seeds in grams	0.95
	plant type	404	Binary	Historical plant architecture data coded as vining or bush	NA
	plant type2	292	Binary	Contemporary plant architecture data coded as vining or bush	NA
	max vig	413	Ordinal	Maximum plant vigor on 1-5 scale	0.588
	min vig	414	Ordinal	Maximum plant vigor on 1-5 scale	0.618
	max width	413	Quantitative	Maximum fruit width in centimeters	0.937
	width min	304	Quantitative	Minimum fruit width in centimeters	1
	len max	413	Quantitative	Maximum fruit length in centimeters	0.748
	len min	315	Quantitative	Minimum fruit length in centimeters	0.841
	len min	421	Ordinal	Maximum fruit thickness in centimeters	0.614
	flesh min	175	Ordinal	Minimum fruit thickness in centimeters	0.425
	sb nymph	205	Quantitative	Number of squash bug nymphs on plan	0.181
	sb adult	249	Quantitative	Number of adult squash bugs on plant	0.206
	cuc inj	247	Ordinal	Severity of beetle damage on a 0-4 scale	0.228
	or flesh	378	Binary	Flesh color coded as orange or not orange	NA
	yl flesh	378	Binary	Flesh color coded as yellow or not yellow	NA
	yl fruit	182	Binary	Color of fruit coded as yellow or not yellow	NA
	tan fruit	182	Binary	Color of fruit coded as tan or not tan	NA
	gn fruit	182	Binary	Color of fruit coded as green or not green	NA
	globe fruit	333	Binary	Fruit shape as globe or not globe	NA
	oblong fruit	333	Binary	Fruit shape as oblong or not oblong	NA
	smooth fruit	130	Binary	Furit texture as smooth or not smooth	NA
	rib fruit	130	Binary	Degree of ribbing	NA
	spec fruit	248	Binary	Fruit patterning as speckled or not speckled	NA
	mot fruit	248	Binary	Fruit patterning as mottled or not mottled	NA
	solid fruit	248	Binary	Fruit patterning as solid color or patterned	NA
***C. moschata***					
	fruit len	123	Quantitative	Fruit length in centimeters	0.804
	fruit diam	123	Quantitative	Fruit diameter in centimeters	0.478
	fruit diam	109	Binary	Fruit maturity as early or late	NA
	or fruit	145	Binary	Fruit color coded as orange or not orange	NA
	smooth fruit	130	Binary	Fruit surface texture encoded as smooth or not smooth	NA
***C. maxima***					
	len	346	Quantitative	Fruit length in centimeters	0.374
	set	350	Ordinal	Fruit set from poor to excellent (1-9)	0.254
	diam	345	Quantitative	Fruit diameter in centimeters	0.249
	watermelon mosaic	297	Ordinal	Susceptibility to WMV from slight to severe (0-9)	0.18
	cuc mosaic	100	Ordinal	Cucumber mosaic susceptibility from slight to severe (0-9)	0.129
	maturity	329	Quantitative	Number of days from field transplanting to date of first pollination	0.388
	unif	341	Ordinal	Fruit uniformity from poor to excellent (1-9)	0.157
	pm	287	Ordinal	Susceptibility to PM from slight to severe (0-9)	0.192
	plant habit	352	Binary	Plant type as vining or not vining	NA
	vig	353	Ordinal	Plant vigor from poor to excellent (1-9)	0.066
	or flesh	288	Binary	Flesh color as orange or not orange	NA
	rib	338	Ordinal	Fruit ribbing from slight to pronounced (1-9)	0.427
	fruit spot	272	Ordinal	Fruit spotting from slight to pronounced (1-9)	0.132
	gray fruit	264	Binary	Fruit color encoded as gray or not gray	NA
	or fruit	264	Binary	Fruit color encoded as orange or not orange	NA
	gn fruit	264	Binary	Fruit color encoded as green or not green	NA

NA, not applicable.

### 3.5 Genome-wide association and synteny analysis

Genome-wide association studies were conducted for all traits using a standard mixed-model K + Q analysis. A weak signal was detected in *C. moschata* on chromosome 3 for fruit length. Weak signals were detected in *C. maxima* for fruit ribbing on chromosome 17 and green fruit on chromosome 20. Five phenotypes were significantly associated with SNPs in *C. pepo*: bush/vine plant architecture on chromosome 10 using contemporary and historic data, fruit flesh thickness on chromosome 2, green fruit on chromosomes 2 and 19, and a non-significant, but clear signal for flesh color on chromosome 5. Weaker associations are shown in **Supplemental Figure S3** with corresponding qqplots in **Figure S4**. The top five SNPs associated with each trait are shown in **Supplemental Table S1**.

**Figure 7:**
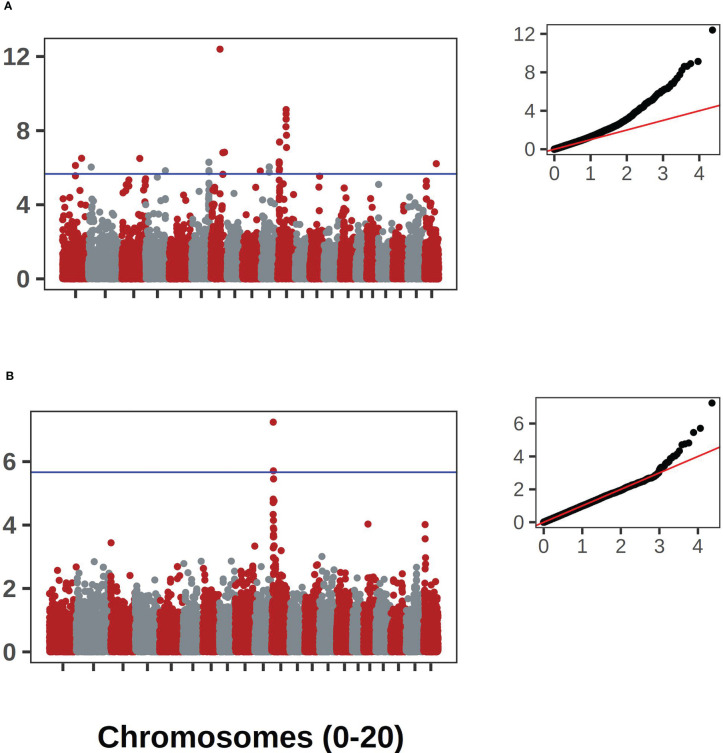
GWAS results for the *Bu* gene in *C. pepo*. **(A)** shows the association results using the historical data with accompanying Q-Q plot. **(B)** shows the results using the contemporary data set. Both analyses supported an association on chromosome 10.

The bush/vine phenotype in *C. pepo* exhibited the strongest signal. The signal was present in both the historical and contemporary data. This historical data consisted of 404 records and the contemporary data had 292 records. The two data sets overlapped by 92 accession records. Manhattan plots for the *Bu* gene GWAS results are shown in **Figure 7A**. Along with corresponding qqplots in **Figure 7B**. The genomic region corresponding to the signal was extracted and used for comparison against the candidate gene for dwarfism in *C. maxima*, CmaCh03G013600. The gene Cp4.1LG10g05740 on chromosome 10 in *C. pepo* was found to be orthologous to CmaCh03G013600 and coincides with the region significantly associated with the bush/vine plant architecture phenotype identified by GWAS in the *C. pepo* collection.

### 3.6 Development of a core collection

**Figure 8:**
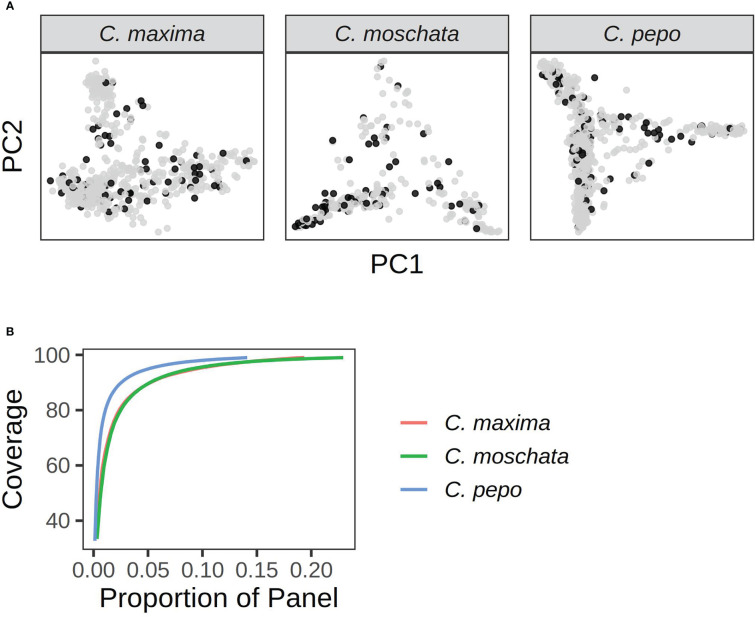
Results from running GenoCore in each of the panels. **(A)** shows the PCA plots for each panel with accessions selected by GenoCore represented as black points. **(B)** shows the proportion of total accessions needed to obtain a certain coverage of diversity.

A core set of accessions that covered over 99% of total genetic diversity was identified in each of the panels. Roughly 5%-10% of the accessions were required to capture the genetic diversity in the panels (see **Figure 8**). This amounted to 117 accessions in *C. pepo*, 72 in *C. moschata*, and 72 in *C. maxima*.

## 4 Discussion

*Cucurbita pepo*, *C. moschata*, and *C. maxima* exhibit a wide range of phenotypic diversity. This diversity is evident in the GRIN phenotypic records for these species. We have demonstrated that there is also a wide range of genetic diversity through genotyping-by-sequencing and genetic analysis of available specimens from the germplasm collections. Thousands to tens of thousands of whole-genome markers where discovered for each species. Clustering of samples and admixture analysis produced results that align closely with known secondary centers of origin in all species. This was especially clear in our analysis of the *C. pepo* collection. *Cucurbita pepo* has its origin in the New World, with a secondary center of diversification in Europe. This pattern was conspicuous in our PCA. Analysis of the admixture patterns within common market classes mirrored the results of the broader diversity panel. For example, it is well known that the Acorn, Scallop and Crook type *C. pepo* were primarily developed in the Americas, whereas Zucchini, Marrow, and Gem squash were developed in Europe. Thus, it is not surprising that Acorn, Scallop, and Crook types have a large proportion of subsp. *ovifera* in their background. Likewise, the Neck, Cheese, and Calabaza types have their origins in the Americas, whereas the Japonica type has more shared ancestry with Asian landraces. The various *C. maxima* market classes were less distinct from one another. Morphologically, many of the classes (Buttercup, Kabocha, and Kuri) are very similar, so it is not surprising that their admixture proportions are similar.

Linkage decay curves showed a common pattern across all species, with the correlation between markers falling off precipitously around 2 Mb. Relative to the other two species, *C. pepo* had a higher baseline LD. This is likely due to the presence of two distinct subspecies, subsp. *ovifera* and subsp. *pepo*, in the *C. pepo* panel. In general, the three *Cucurbita* species studied have much higher LD than other outcrosses, such as maize. Studies in maize have shown that LD drops off within kilobases rather than megabases in diverse accessions (Yan et al., 2009). This suggests that the effective population size of *Cucurbita* species is much smaller than other agricultural species, and is consistent with studies looking at smaller panels in *Cucurbita* (Xanthopoulou et al., 2019). Although we have fewer markers in *C. maxima*, it is likely that the number of markers is sufficient to pick up major population structure in the panel given the extent of LD and clear results observed in the PCA.

Our GWAS analysis using contemporary and historic plant habit data led to the mapping of a locus on chromosome 10 associated with the bush/vine phenotype. It is notable that the contemporary and historical data were on different accessions, overlap of less than half. These associations represent validation using two distinct panels. This locus is likely the bush gene (*Bu*) locus that has been finely mapped to this location in previous *C. pepo* studies (Xiang et al., 2018; Ding et al., 2021). Although our GWAS hit does not constitute a novel gene association, it does demonstrate that the *Bu* locus, previously mapped in biparental populations, is also the primary driver of the bush phenotype in diverse germplasm. Thus, this locus is likely to have utility across a wide array of germplasm. We also demonstrated that this locus is syntenic with the bush gene previously mapped in *C. maxima* (Zhang et al., 2015). Recent work has also identified a bush gene in *C. moschata*, and underscores the importance of this trait for productivity in cucurbits (Wang et al., 2022). There are many other developmental and morphological traits shared across *Cucurbita* (Paris and Brown, 2005). Our results demonstrate the power of leveraging information across species within *Cucurbita*, and suggests the potential of transferring knowledge from the more studied *C. pepo* to *C. moschata* and *C. maxima*.

Few clear signals were detected for traits outside of plant habit in *C. pepo*. The goal of the USDA GRIN collection is to maintain genetic diversity, not necessarily true breeding stocks. Given that each species is out-crossing, there is inevitably heterogeneity in stocks. Heterogeneity was undoubtedly a complicating factor in our study. There would be a great benefit from phenotyping and genotyping stocks purified from the USDA collection; however, such an experiment was well outside the scope of this study. A further complicating factor of GWAS is trait architecture. Traits with a more complex architecture are not amenable to GWAS analysis, as complex traits are often governed by many loci of small effect. These traits are better targets for prediction using genome-wide markers (Meuwissen et al., 2001). We accessed the ability of whole-genome markers to capture trait variability by calculating genomic heritability for all quantitative and ordinal traits. These estimates were high for many of the morphological and agronomic traits in each species. Yet, no major loci were detected for these same traits *via* GWAS. This points towards these traits having a more complex trait architecture. The moderate to high genomic heritability observed for morphological traits in this study is consistent with other estimates in squash (Hernandez et al., 2020).

High genomic heritability estimates with no significant association is a hallmark of more complex traits. A complex trait architecture is not the only explanation though. Confounding of a phenotype with population structure can lead to a similar outcome—the K + Q model will remove the association, but the genomic heritability will remain high. We observed that seed weight in *C. pepo*, maturity in *C. moschata*, and plant habit in *C. maxima* were strongly associated with population (see **Figure 6**). Association of plant habit with population structure in *C. maxima* helps explain why we were unable to recapitulate the known major effect *Bu* locus. A good approach for future studies hoping to elucidate loci underlying these traits with the germplasm panels presented would be to form biparental or multiparental populations across genetic groups to break up structure. A similar approach was used to map genes related to cucurbitacin content associated with subspecies in *C. pepo* (Brzozowski et al., 2020).

Our data provides many genome-wide markers which could be used as a source of markers to develop marker panels for use in breeding applications, as has been done in other crops (Arbelaez et al., 2019). Possible breeding applications would include marker assisted selection, marker assisted backcrossing, and purity assessment of seedstock using a low density panel; whereas, a medium density panel could be developed for routine genomic selection (Cerioli et al., 2022). Our clustering of samples based on marker data suggest geography is a key driver for overall population structure. When projecting ancestry proportions onto cultivars of known market classes, the ancestry proportions were relatively similar within market class grouping. Although there is genetic diversity within each species, this diversity is constrained within market classes. This suggests that crosses between these market classes would greatly increase the amount of genetic diversity to be leveraged in breeding efforts. Crossing between market classes would come at the cost of bringing in undesirable characteristics with regard to achieving a specific morphotype associated market class. This cost could be mitigated through the use of markers to recover morphotype expeditiously during pre-breeding (Cobb et al., 2019).

Our data provides a useful starting point for future studies. In the case where traits are common in the panel, the panel can be phenotyped for a trait of interest and combined with marker data and insight provided by our study. We demonstrated this approach in our association analysis of the bush gene. In the case of a rare phenotype, such as a resistance gene, subsets of the germplasm and markers should be used to develop custom populations. Plant introductions (PI) are frequently used as source parents in mapping studies and for germplasm improvement, as was the case for mapping Phytophthora capsici resistance and developing resistant breeding lines (LaPlant et al., 2020; Vogel et al., 2021). We found some traits that had high heritability, such as morphological traits, but we were not able to find any associations. Genomic predication rather than association may be the best approach for these traits. In other cases, it may be required to break population structure through crossing as we observed with seed weight in *C. pepo*, maturity in *C. moschata*, and plant habit it *C. maxima*. Certain applications, such as the creation of a hapmap or diversity atlas, require higher density re-sequencing data. Our GenoCore analysis provides subsets that will be useful in these efforts.

## Data availability statement

The datasets presented in this study can be found in online repositories. The names of the repository/repositories and accession number(s) can be found in the article/**Supplementary Material**.

## Author contributions

CH wrote the first draft. MM, ZF, and RG provided project oversight. CH, JF, and KB conducted data analysis. KR and JL assisted with data curation and germplasm selection. MM, RG and ZF designed the experiment. JL contributed data. All authors contributed to the article and approved the submitted version.

## References

[B1] Alexander D. H. Lange K. (2011). Enhancements to the ADMIXTURE algorithm for individual ancestry estimation. BMC Bioinf. 12, 1–6. doi: 10.1186/1471-2105-12-246 PMC3146885 21682921

[B2] Arbelaez J. D. Dwiyanti M. S. Tandayu E. Llantada K. Jarana A. Ignacio J. C.. (2019). 1k-RiCA (1K-rice custom amplicon) a novel genotyping amplicon-based SNP assay for genetics and breeding applications in rice. Rice 12, 55. doi: 10.1186/s12284-019-0311-0 31350673 PMC6660535

[B3] Bradbury P. J. Zhang Z. Kroon D. E. Casstevens T. M. Ramdoss Y. Buckler E. S. (2007). TASSEL: software for association mapping of complex traits in diverse samples. Bioinformatics 23, 2633–2635. doi: 10.1093/bioinformatics/btm308 17586829

[B4] Brzozowski L. J. Gore M. A. Agrawal A. A. Mazourek M. (2020). Divergence of defensive cucurbitacins in independent domestication events leads to differences in specialist herbivore preference. Plant Cell Environ. 43, 2812–2825. doi: 10.1111/pce.13844 32666553

[B5] Cerioli T. Hernandez C. O. Angira B. McCouch S. R. Robbins K. R. Famoso A. N. (2022). Development and validation of an optimized marker set for genomic selection in southern U.S. rice breeding programs. Plant Genome 15, e20219. doi: 10.1002/tpg2.20219 35611838 PMC12806970

[B6] Chomicki G. Schaefer H. Renner S. S. (2020). Origin and domestication of cucurbitaceae crops: insights from phylogenies, genomics and archaeology. New Phytol. 226, 1240–1255. doi: 10.1111/nph.16015 31230355

[B7] Cobb J. N. Biswas P. S. Platten J. D. (2019). Back to the future: revisiting MAS as a tool for modern plant breeding. Theor. Appl. Genet. 132, 647–667. doi: 10.1007/s00122-018-3266-4 30560465 PMC6439155

[B8] Danecek P. Auton A. Abecasis G. Albers C. A. Banks E. DePristo M. A.. (2011). The variant call format and VCFtools. Bioinformatics 27, 2156–2158. doi: 10.1093/bioinformatics/btr330 21653522 PMC3137218

[B9] Danecek P. Bonfield J. K. Liddle J. Marshall J. Ohan V. Pollard M. O.. (2021). Twelve years of SAMtools and BCFtools. GigaScience 10, giab008. doi: 10.1093/gigascience/giab008 33590861 PMC7931819

[B10] de los Campos G. Sorensen D. Gianola D. (2015). Genomic heritability: What is it? PloS Genet. 11, e1005048. doi: 10.1371/journal.pgen.1005048 25942577 PMC4420472

[B11] Ding W. Wang Y. Qi C. Luo Y. Wang C. Xu W.. (2021). Fine mapping identified the gibberellin 2-oxidase gene CpDw leading to a dwarf phenotype in squash (cucurbita pepo l.). Plant Sci. 306, 110857. doi: 10.1016/j.plantsci.2021.110857 33775356

[B12] Endelman J. B. (2011). Ridge regression and other kernels for genomic selection with r package rrBLUP. Plant Genome 4, 250–255. doi: 10.3835/plantgenome2011.08.0024

[B13] Ferriol M. Picó B. (2008). “Pumpkin and winter squash,” in Handbook of plant breeding (Berlin: Springer New York), 317–349. doi: 10.1007/978-0-387-30443-4_10

[B14] Glaubitz J. C. Casstevens T. M. Lu F. Harriman J. Elshire R. J. Sun Q.. (2014). TASSEL-GBS: A high capacity genotyping by sequencing analysis pipeline. PloS One 9, e90346. doi: 10.1371/journal.pone.0090346 24587335 PMC3938676

[B15] Gogarten S. M. Sofer T. Chen H. Yu C. Brody J. A. Thornton T. A.. (2019). Genetic association testing using the GENESIS r/bioconductor package. Bioinformatics 35, 5346–5348. doi: 10.1093/bioinformatics/btz567 31329242 PMC7904076

[B16] Grumet R. McCreight J. D. McGregor C. Weng Y. Mazourek M. Reitsma K.. (2021). Genetic resources and vulnerabilities of major cucurbit crops. Genes 12, 1222. doi: 10.3390/genes12081222 34440396 PMC8392200

[B17] Hernandez C. O. Wyatt L. E. Mazourek M. R. (2020). Genomic prediction and selection for fruit traits in winter squash. G3 Genes|Genomes|Genetics 10, 3601–3610. doi: 10.1534/g3.120.401215 32816923 PMC7534422

[B18] Jeong S. Kim J.-Y. Jeong S.-C. Kang S.-T. Moon J.-K. Kim N. (2017). GenoCore: A simple and fast algorithm for core subset selection from large genotype datasets. PloS One 12, e0181420. doi: 10.1371/journal.pone.0181420 28727806 PMC5519076

[B19] Kates H. R. Soltis P. S. Soltis D. E. (2017). Evolutionary and domestication history of cucurbita (pumpkin and squash) species inferred from 44 nuclear loci. Mol. Phylogenet. Evol. 111, 98–109. doi: 10.1016/j.ympev.2017.03.002 28288944

[B20] Kaźmińska K. Hallmann E. Rusaczonek A. Korzeniewska A. Sobczak M. Filipczak J.. (2018). Genetic mapping of ovary colour and quantitative trait loci for carotenoid content in the fruit of cucurbita maxima duchesne. Mol. Breed. 38, 114. doi: 10.1007/s11032-018-0869-z 30237748 PMC6133072

[B21] LaPlant K. E. Vogel G. Reeves E. Smart C. D. Mazourek M. (2020). Performance and resistance to phytophthora crown and root rot in squash lines. HortTechnology 30, 608–618. doi: 10.21273/horttech04636-20

[B22] Li H. Durbin R. (2009). Fast and accurate short read alignment with burrows-wheeler transform. Bioinformatics 25, 1754–1760. doi: 10.1093/bioinformatics/btp324 19451168 PMC2705234

[B23] Loy J. B. (2004). Morpho-physiological aspects of productivity and quality in squash and pumpkins (cucurbita spp.). Crit. Rev. Plant Sci. 23, 337–363. doi: 10.1080/07352680490490733

[B24] Lust T. A. Paris H. S. (2016). Italian Horticultural and culinary records of summer squash (cucurbita pepo, cucurbitaceae) and emergence of the zucchini in 19th-century milan. Ann. Bot. 118, 53–69. doi: 10.1093/aob/mcw080 27343231 PMC4934399

[B25] McCouch S. Navabi Z. K. Abberton M. Anglin N. L. Barbieri R. L. Baum M.. (2020). Mobilizing crop biodiversity. Mol. Plant 13, 1341–1344. doi: 10.1016/j.molp.2020.08.011 32835887

[B26] Meuwissen T. H. E. Hayes B. J. Goddard M. E. (2001). Prediction of total genetic value using genome-wide dense marker maps. Genetics 157, 1819–1829. doi: 10.1093/genetics/157.4.1819 11290733 PMC1461589

[B27] Money D. Gardner K. Migicovsky Z. Schwaninger H. Zhong G.-Y. Myles S. (2015). LinkImpute: Fast and accurate genotype imputation for nonmodel organisms. G3 Genes|Genomes|Genetics 5, 2383–2390. doi: 10.1534/g3.115.021667 26377960 PMC4632058

[B28] Montero-Pau J. Blanca J. Esteras C. Martínez-Pérez E. M. Gómez P. Monforte A. J.. (2017). An SNP-based saturated genetic map and QTL analysis of fruit-related traits in zucchini using genotyping-by-sequencing. BMC Genomics 18, 94. doi: 10.1186/s12864-016-3439-y 28100189 PMC5241963

[B29] Nee M. (1990). The domestication ofcucurbita (cucurbitaceae). Economic Bot. 44, 56–68. doi: 10.1007/bf02860475

[B30] Paris H. S. (2015). Germplasm enhancement of cucurbita pepo (pumpkin, squash, gourd: Cucurbitaceae): progress and challenges. Euphytica 208, 415–438. doi: 10.1007/s10681-015-1605-y

[B31] Paris H. S. Brown R. N. (2005). The genes of pumpkin and squash. HortScience 40, 1620–1630. doi: 10.21273/hortsci.40.6.1620

[B32] Paris H. S. Lebeda A. Křistkova E. Andres T. C. Nee M. H. (2012). Parallel evolution under domestication and phenotypic differentiation of the cultivated subspecies of cucurbita pepo (cucurbitaceae). Economic Bot. 66, 71–90. doi: 10.1007/s12231-012-9186-3

[B33] Purcell S. Neale B. Todd-Brown K. Thomas L. Ferreira M. A. Bender D.. (2007). PLINK: A tool set for whole-genome association and population-based linkage analyses. Am. J. Hum. Genet. 81, 559–575. doi: 10.1086/519795 17701901 PMC1950838

[B34] Savage J. A. Haines D. F. Holbrook M. N. (2015). The making of giant pumpkins: how selective breeding changed the phloem of cucurbita maxima from source to sink. Plant Cell Environ. 38, 1543–1554. doi: 10.1111/pce.12502 25546629

[B35] Sun H. Wu S. Zhang G. Jiao C. Guo S. Ren Y.. (2017). Karyotype stability and unbiased fractionation in the paleo-allotetraploid cucurbita genomes. Mol. Plant 10, 1293–1306. doi: 10.1016/j.molp.2017.09.003 28917590

[B36] Vogel G. LaPlant K. E. Mazourek M. Gore M. A. Smart C. D. (2021). A combined BSA-seq and linkage mapping approach identifies genomic regions associated with phytophthora root and crown rot resistance in squash. Theor. Appl. Genet. 134, 1015–1031. doi: 10.1007/s00122-020-03747-1 33388885

[B37] Wang X. Ando K. Wu S. Reddy U. K. Tamang P. Bao K.. (2021). Genetic characterization of melon accessions in the u.s. national plant germplasm system and construction of a melon core collection. Mol. Horticult. 1, 1–3. doi: 10.1186/s43897-021-00014-9 PMC10515074 37789496

[B38] Wang X. Bao K. Reddy U. K. Bai Y. Hammar S. A. Jiao C.. (2018). The USDA cucumber (cucumis sativus l.) collection: genetic diversity, population structure, genome-wide association studies, and core collection development. Horticult. Res. 5. doi: 10.1038/s41438-018-0080-8 PMC6165849 30302260

[B39] Wang S. Wang K. Li Z. Li Y. He J. Li H.. (2022). Architecture design of cucurbit crops for enhanced productivity by a natural allele. Nat. Plants 8, 1394–1407. doi: 10.1038/s41477-022-01297-6 36509843

[B40] Wu P.-Y. Tung C.-W. Lee C.-Y. Liao C.-T. (2019a). Genomic prediction of pumpkin hybrid performance. Plant Genome 12, 180082. doi: 10.3835/plantgenome2018.10.0082 PMC12810009 31290920

[B41] Wu S. Wang X. Reddy U. Sun H. Bao K. Gao L.. (2019b). Genome of ‘charleston gray’, the principal american watermelon cultivar, and genetic characterization of 1,365 accessions in the u.s. national plant germplasm system watermelon collection. Plant Biotechnol. J. 17, 2246–2258. doi: 10.1111/pbi.13136 31022325 PMC6835170

[B42] Xanthopoulou A. Montero Pau J. Mellidou I. Kissoudis C. Blanca J. Picó B.. (2019). Whole-genome resequencing of cucurbita pepo morphotypes to discover genomic variants associated with morphology and horticulturally valuable traits. Horticult. Res. 6, 94. doi: 10.1038/s41438-019-0176-9 PMC6804688 31645952

[B43] Xiang C. Duan Y. Li H. Ma W. Huang S. Sui X.. (2018). A high-density EST-SSR-based genetic map and QTL analysis of dwarf trait in cucurbita pepo l. Int. J. Mol. Sci. 19, 3140. doi: 10.3390/ijms19103140 30322052 PMC6213718

[B44] Yan J. Shah T. Warburton M. L. Buckler E. S. McMullen M. D. Crouch J. (2009). Genetic characterization and linkage disequilibrium estimation of a global maize collection using SNP markers. PloS One 4, e8451. doi: 10.1371/journal.pone.0008451 20041112 PMC2795174

[B45] Yu J. Wu S. Sun H. Wang X. Tang X. Guo S.. (2023). CuGenDBv2: an updated database for cucurbit genomics. Nucleic Acids Res 51 (D1), D1457–D1464. doi: 10.1093/nar/gkac921 PMC9825510 36271794

[B46] Zhang G. Ren Y. Sun H. Guo S. Zhang F. Zhang J.. (2015). A high-density genetic map for anchoring genome sequences and identifying QTLs associated with dwarf vine in pumpkin (Cucurbita maxima duch.). BMC Genomics 16, 1101. doi: 10.1186/s12864-015-2312-8 26704908 PMC4690373

[B47] Zheng X. Levine D. Shen J. Gogarten S. M. Laurie C. Weir B. S. (2012). A high-performance computing toolset for relatedness and principal component analysis of SNP data. Bioinformatics 28, 3326–3328. doi: 10.1093/bioinformatics/bts606 23060615 PMC3519454

[B48] Zhong Y.-J. Zhou Y.-Y. Li J.-X. Yu T. Wu T.-Q. Luo J.-N.. (2017). A high-density linkage map and QTL mapping of fruit-related traits in pumpkin (Cucurbita moschata duch.). Sci. Rep. 7, 12785. doi: 10.1038/s41598-017-13216-3 28986571 PMC5630576

