# Supplementary material for: Characterization of the USDA Cucurbita pepo, C. moschata, and C. maxima germplasm collections
Source: Front Plant Sci. 2023 Mar 13;14:1130814. doi: 10.3389/fpls.2023.1130814 (PMC10040574; doi:10.3389/fpls.2023.1130814)
Supplement: Supplementary file 1 [file DataSheet_1.pdf]

Supplementary Material

1 SUPPLEMENTARY DATA

Raw sequencing data and SNP sets for each species can be found at http://cucurbitgenomics.org/v2/ .

1.1 Figures

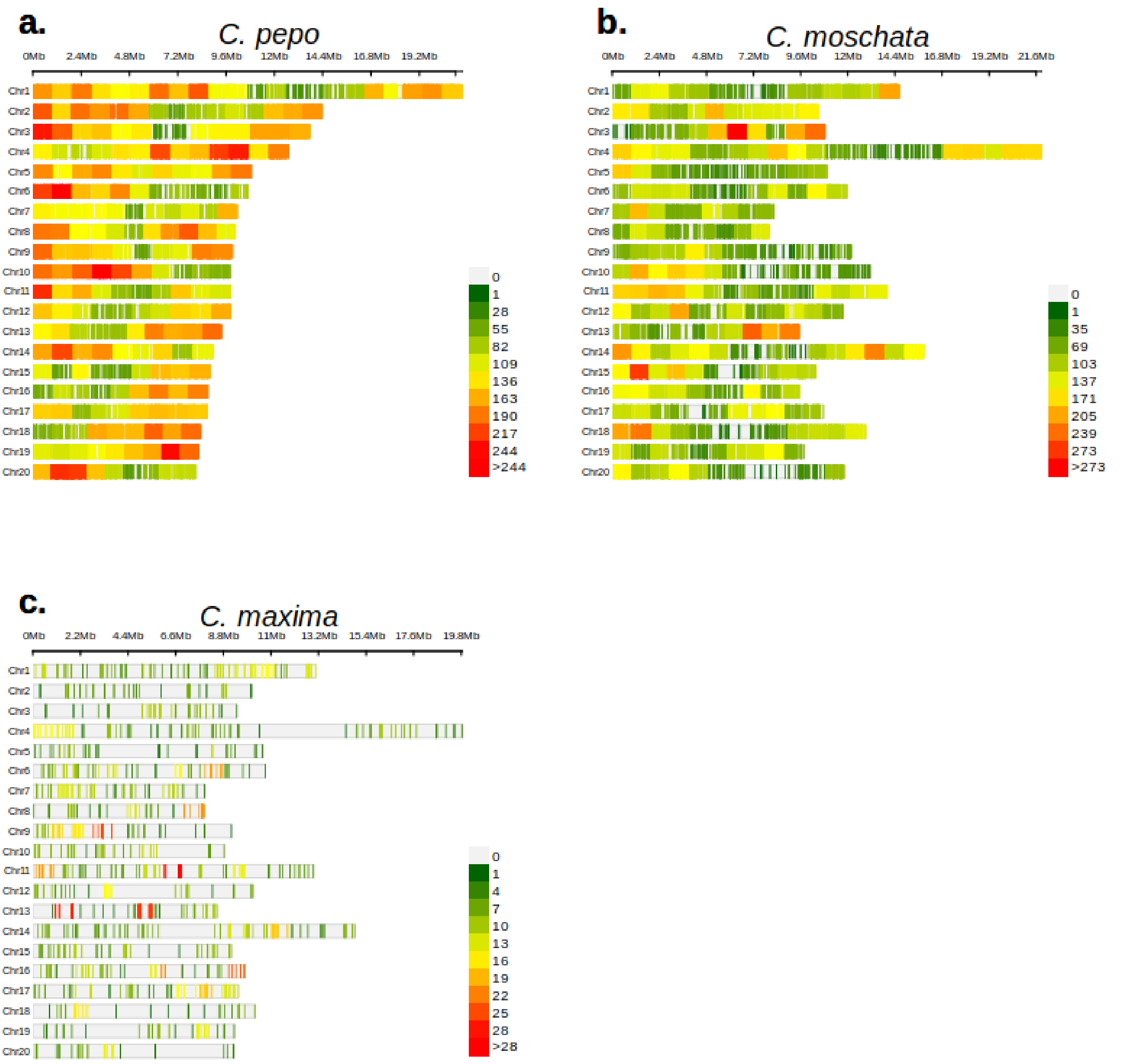

Figure S1. SNP distribution across chromosomes for **Panel a.** *C. pepo*, **Panel b.** *C. moschata*, and **Panel c.** *C. maxima*.

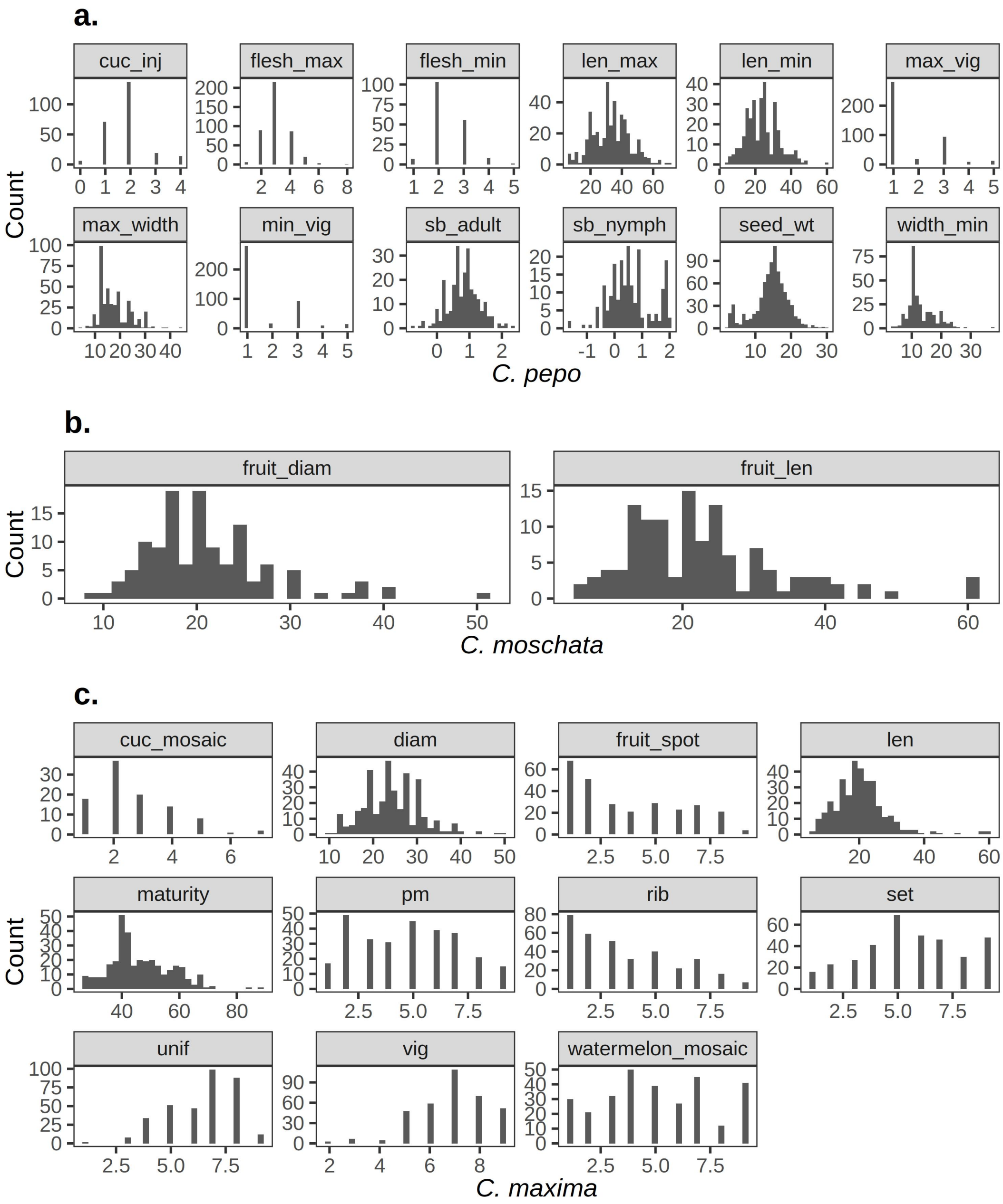

Figure S2. Histograms of continuous and ordinal traits for **Panel a.** *C. pepo*, **Panel b.** *C. moschata*, and **Panel c.** *C. maxima.*

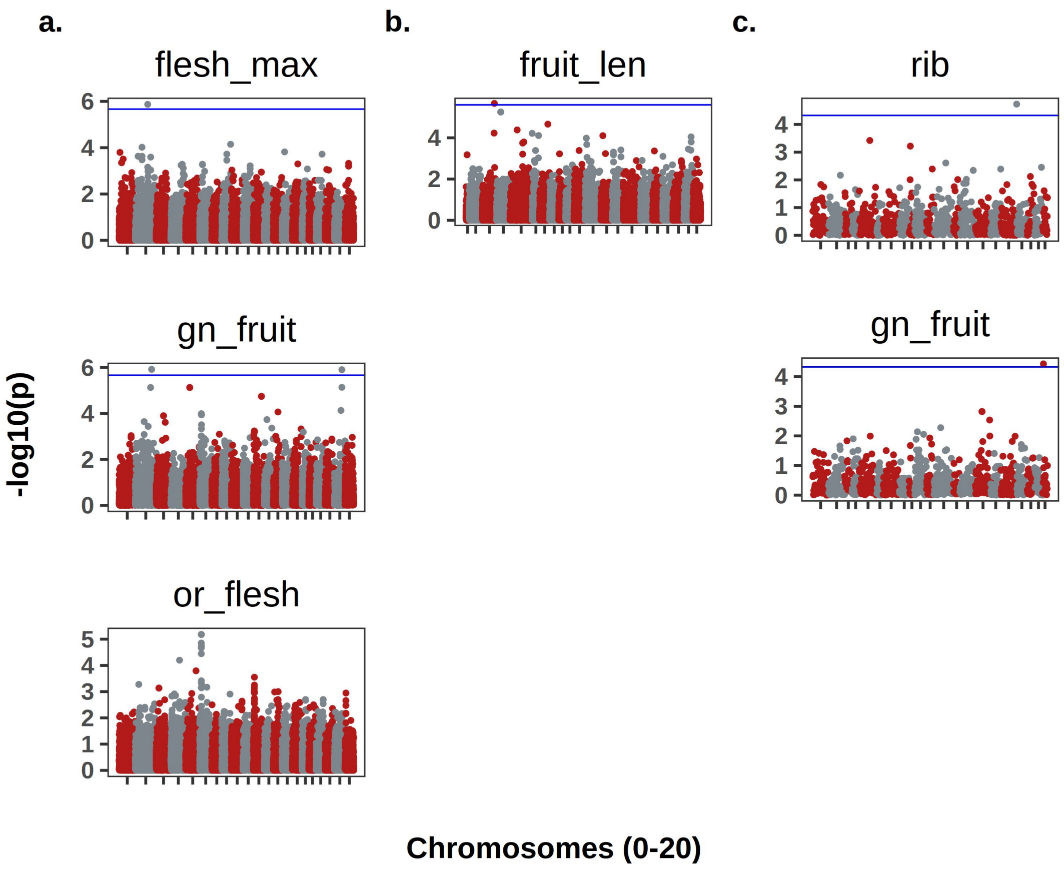

Figure S3. GWAS results for **Panel a.** *C. pepo*, **Panel b.** *C. moschata*, and **Panel c.** *C. maxima.*

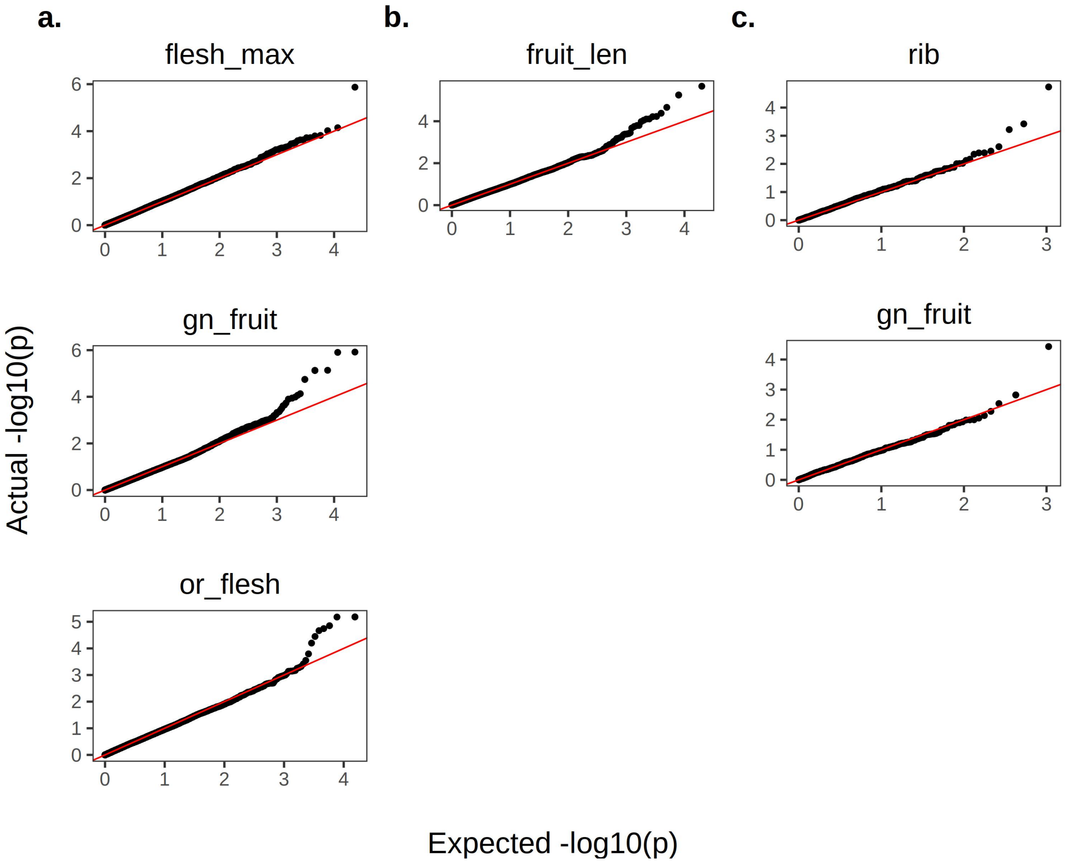

Figure S4. Q-Q plots for GWAS results for **Panel a.** *C. pepo*, **Panel b.** *C. moschata*, and **Panel c.** *C. maxima.*

1.2 Tables

Trait	Species	Chrom	Pos	Alleles
plant_type	*C. pepo*	6	3962527	G/C
		10	3507098	C/T
		10	3507179	G/T
		10	3507087	A/G
		10	3507387	C/T
plant_type2	*C. pepo*	10	679593	A/C
		10	753000	T/C
		10	768265	T/C
		10	666810	T/C
		10	974041	G/A
gn_fruit	*C. pepo*	1	17395123	C/T
		19	6509248	A/G
		19	6509202	T/C
		1	16658797	G/C
		4	5142030	C/T
or_flesh	*C. pepo*	5	1075625	A/T
		5	1075652	T/G
		5	1074883	T/C
		5	1072306	G/T
		5	1115573	C/T
flesh_max	*C. pepo*	1	10063336	C/T
		7	9444225	T/A
		1	5697024	C/T
		13	2248185	G/A
		0	1463850	G/A
fruit_len	*C. moschata*	2	9116220	T/C
		3	5539269	T/A
		3	5539270	T/A
		6	10461612	C/T
		4	6338885	C/T
rib	*C. maxima*	17	76012	T/C
		4	8332568	C/T
		8	1759117	A/T
		11	6776288	A/G
		19	8123733	A/C
gn_fruit	*C. maxima*	20	1087480	G/A
		14	9051301	G/T
		14	9051304	A/T
		14	14410557	C/T
		11	3829766	C/T

Table S1. The position and allele are shown for the top five association results for each trait for which there was a GWAS signal.
